# Supplementary material for: From sequence to enzyme mechanism using multi-label machine learning
Source: BMC Bioinformatics. 2014 May 19;15:150. doi: 10.1186/1471-2105-15-150 (PMC4229970; doi:10.1186/1471-2105-15-150)
Supplement: Additional file 2 — Java code of ml2db. Additional file ml2db_code.tar.gz contains the Java source code to run the multi-label machine learning experiments and save the results to database. The code’s Javadoc is included. [file 1471-2105-15-150-S2.zip › additional file 2/ml2db/ml2db/doc/mulan/core/MulanJavadoc.html]

MulanJavadoc


JavaScript is disabled on your browser.


- Overview
- Package
- Class
- Use
- Tree
- Deprecated
- Index
- Help

- Prev Class
- Next Class

- Frames
- No Frames

- All Classes

- Summary:
- Nested |
- Field |
- Constr |
- Method

- Detail:
- Field |
- Constr |
- Method


mulan.core

## Class MulanJavadoc

- java.lang.Object
- - mulan.core.MulanJavadoc

- ---

    

  ```
  public class MulanJavadoc
  extends java.lang.Object
  ```

  This class uses weka's Javadoc auto-generation classes to generate Javadoc

  Version:
  :   2012.02.27

  Author:
  :   Eleftherios Spyromitros-Xioufis, Grigorios Tsoumakas

- - ### Constructor Summary

    Constructors

    Constructor and Description

    `MulanJavadoc()`
  - ### Method Summary

    Methods

    | Modifier and Type | Method and Description |
    | --- | --- |
    | `java.lang.String` | `globalInfo()` Returns global information about the class |
    | `static void` | `main(java.lang.String[] args)` Command line interface |
    | `static void` | `recursiveTraversal(java.io.File fileObject)` Recursively visit all files |
    | `static void` | `setDir(java.lang.String dir)` Sets the direcrory |
    | `static void` | `traverse()` Recursively visit all files |
    | `static void` | `updateJavadoc(java.lang.String classname)` Updates comments |

    - ### Methods inherited from class java.lang.Object

      `equals, getClass, hashCode, notify, notifyAll, toString, wait, wait, wait`

- - ### Constructor Detail


    - #### MulanJavadoc

      ```
      public MulanJavadoc()
      ```
  - ### Method Detail


    - #### setDir

      ```
      public static void setDir(java.lang.String dir)
      ```

      Sets the direcrory

      Parameters:
      :   `dir` -


    - #### main

      ```
      public static void main(java.lang.String[] args)
                       throws java.lang.Exception
      ```

      Command line interface

      Parameters:
      :   `args` -

      Throws:
      :   `java.lang.Exception`


    - #### updateJavadoc

      ```
      public static void updateJavadoc(java.lang.String classname)
      ```

      Updates comments

      Parameters:
      :   `classname` -


    - #### traverse

      ```
      public static void traverse()
                           throws java.lang.Exception
      ```

      Recursively visit all files

      Throws:
      :   `java.lang.Exception`


    - #### recursiveTraversal

      ```
      public static void recursiveTraversal(java.io.File fileObject)
                                     throws java.lang.Exception
      ```

      Recursively visit all files

      Parameters:
      :   `fileObject` -

      Throws:
      :   `java.lang.Exception`


    - #### globalInfo

      ```
      public java.lang.String globalInfo()
      ```

      Returns global information about the class

      Returns:
      :   global information


- Overview
- Package
- Class
- Use
- Tree
- Deprecated
- Index
- Help

- Prev Class
- Next Class

- Frames
- No Frames

- All Classes

- Summary:
- Nested |
- Field |
- Constr |
- Method

- Detail:
- Field |
- Constr |
- Method
